# Case report: The treatment for olfactory neuroblastoma combined with leptomeningeal carcinomatosis *via* an ommaya reservoir

**DOI:** 10.3389/fonc.2022.1060575

**Published:** 2023-01-13

**Authors:** Yichen Peng, Xun Kang, Bo Jiang, Feng Chen, Shoubo Yang, Zhuang Kang, Ce Wang, Yi Lin, Shenglan Li, Jiefei Han, Botao Zhang, Weichunbai Zhang, Wenbin Li

**Affiliations:** Department of Neuro-oncology, Cancer Center, Beijing Tiantan Hospital, Capital Medical University, Beijing, China

**Keywords:** olfactory neuroblastoma, leptomeningeal carcinomatosis, intrathecal administration, ommaya reservoir, case report

## Abstract

Olfactory neuroblastoma is a rare neoplasm that usually presents in the upper nasal cavity. Although its prognosis is highly unfavorable, effective treatment options are still lacking. Moreover, there is no standard treatment for patients with olfactory neuroblastoma that progressed to leptomeningeal carcinomatosis. Here we report an uncommon case of a 59-year-old woman who was diagnosed with olfactory neuroblastoma and leptomeningeal carcinomatosis. For a direct delivery of the drugs to the tumor, and to avoid the impact of lumbar puncture on the patient’s quality of life, the intravenous chemotherapy plus intrathecal administration of MTX *via* an Ommaya reservoir was chosen. The results were striking, with the disappearance of tumor cells in the cerebrospinal fluid and the relief of the patient’s symptoms with PR. Our result indicates that chemotherapy *via* an Ommaya reservoir offers a new potential therapy for patients with meningeal metastases.

## Introduction

Cancer is a growing global health issue. According to a report from the World Health Organization (WHO), cancer is the first or second leading cause of death for people aged 70 years or less in 112 out of 183 countries and the third or fourth in 23 countries (1). Although many studies investigated head and neck tumors, further investigations are required to better understand the mechanisms and treatments of rare tumors such as olfactory neuroblastoma (ONB). ONB is a rare neoplasm that originates from the olfactory mucosa of the cribriform plate, the nasal septum, and the superior turbinate (2, 3). It can spread to the subarachnoid spaces resulting in high rates of metastasis and recurrence (4). Surgery and radiotherapy are commonly used to treat ONB. It had been reported that the prognosis of surgically treated ONB is significantly better than that of non-surgically treated patients (5). However, the role of chemotherapy remains uncertain. Moreover, leptomeningeal carcinomatosis (LM), which is defined as a malignant invasion of the leptomeninges, caused by the tumor metastatic process, is one of the most uncommon and unfavorable complications of ONB (6). Although the exact mechanism remains unknown, the cerebrospinal fluid (CSF) contacting ONB, and the meninges may be one of the possible participants in the metastasis of leptomeningeal cancer (7).

Unfortunately, due to its low incidence, there are no randomized clinical trials to guide the treatment for ONB, and the standard treatment for ONB with LM is rarely discussed (8). Given the growing unsatisfied condition of the existing treatments to prolong patients’ survival with an acceptable quality of life, a new therapy is desperately needed.

Here, we report a case of a patient with ONB and LM who had rhinorrhea and weakness in both legs when admitted to our hospital. She had a good response to a treatment combination of intravenous chemotherapy and intrathecal injection of methotrexate (MTX). This therapy provided a glimmer of hope for a potentially improved treatment of ONB.

## Patient information and clinical findings

A 59-year-old woman developed sudden but constant rhinorrhea with yellow mucus or occasional blood in January 2020. She denied any family history or mental illness. A month later, she developed hyposmia in addition to the symptoms above. She was evaluated at a local hospital and the brain magnetic resonance imaging (MRI) showed a mass located in the nose and protruded into the skull. This result was confirmed by a rhinoscopy. A grayish-white, infiltrative neoplasm, centered in the inferior meatus and common nasal meatus, was found.

**Figure 1:**
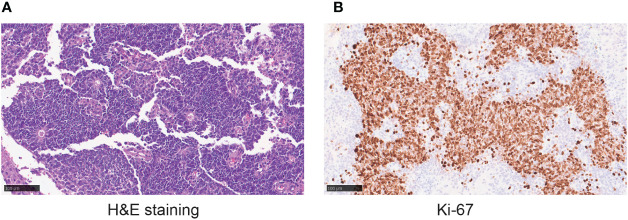
**(A)**The hematoxylin-eosin (H&E) staining in the microscopic observation (20×) showing tumor cells with consistent morphology and size, spindle-shaped nuclei, and a high nucleus to cytoplasm ratio. Flexner-Wintersteiner rosettes can be seen. **(B)** Ki-67 staining in the microscopic observation (20×).

To further diagnose the disease, the patient underwent endoscopic nasal tumor resection, open sinus surgery, skull base repair following endoscopy, and radiofrequency ablation at the local hospital in April 2020. The surgical procedure was successful, and the postoperative pathology confirmed ONB with 90% Ki-67 (**Figure 1**). The patient received radiotherapy with a total dose of 59.36Gy/28F from April to June 2020. She was also treated with 1 cycle of etoposide (200mg, day1-3) and cisplatin (40mg, day1-2) in April 2020. Unfortunately, the treatment was unsuccessful, and the patient’s symptoms remained unimproved.

In July 2020, two weeks before she was admitted to our hospital, her symptoms progressed with a developed and worsening weakness in both lower legs.

## Diagnostic assessment

When the patient was transferred to our hospital in July 2020, she complained that her rhinorrhea was not getting any better compared to 6 months ago, and the weakness in both legs was worsening. On admission, physical examination revealed reduced mental clarity and a lack of speech fluency. She also had impaired orientation, comprehension, calculation, and memory. Cranial nerve testing showed that the pupillary light reflex was dull and that the shoulder shrug was bilaterally eliminated. The grading of power was 2-3 in the right lower limb, 2 in the left lower limb, and 5 in both upper limbs. She also had hypotonia and hyperesthesia, but no abnormalities in proprioceptive sensation.

**Figure 2:**
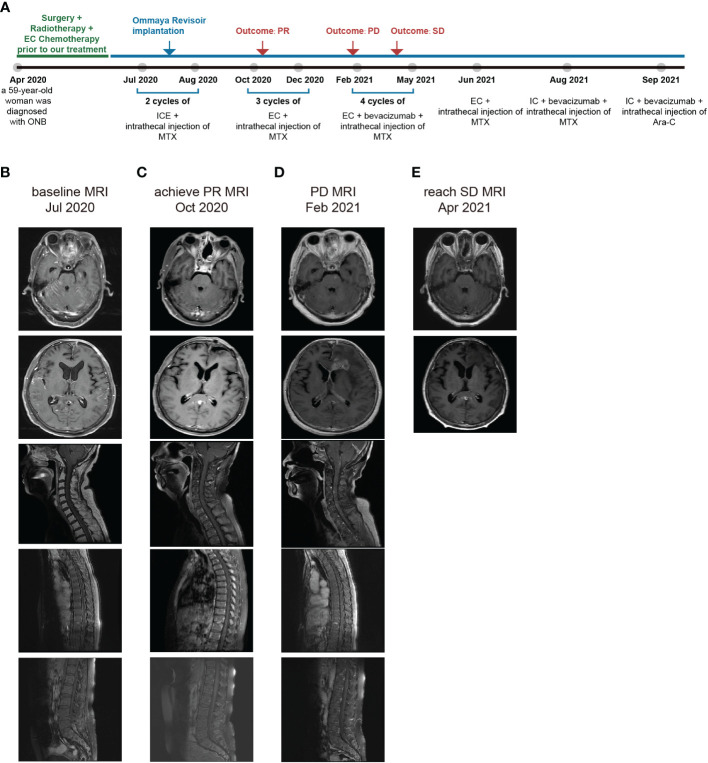
**(A)** Timeline depicting diagnoses, changes in treatment, duration, and outcome. **(B)** MRI at the time admitted to our hospital. **(C)** MRI when achieving the PR. **(D)** MRI at PD. **(E)** MRI when reaching SD.

**Figure 3:**
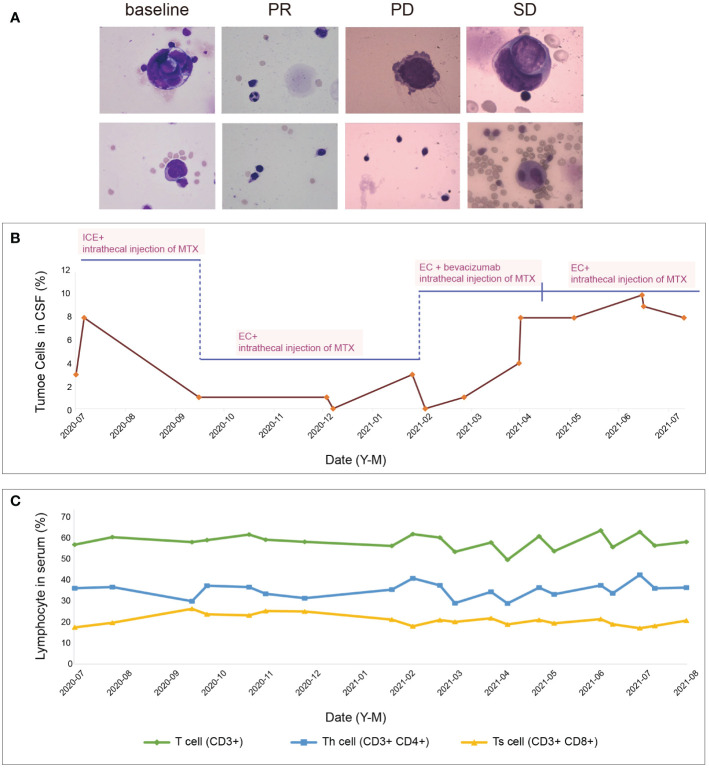
**(A)** Morphological testing of CSF. **(B)** Percentage of tumor cells in cerebrospinal fluid and its correspondence with therapeutic protocols. **(C)** Percentage of T cells in serum and its correspondence with various treatment regimens.

To better evaluate her condition, we performed a series of baseline tests. The MRI revealed abnormal signals in the front lobe, nasal cavity, cervical spinal cord, upper thoracic spinal cord, and lumbar spinal cord (**Figure 2B**). CSF, which nourishes the brain tissue and carries away the metabolites, can relatively reflect the tumor condition in the brain. Correspondingly, the CSF test showing the presence of tumor cells (7%) confirmed the progression of the LM (**Figure 3A**). Based on the patient’s symptoms, physical examination, MRI, and pathology, we concluded that the patient had ONB with Kadish stage D. Kadish D is the most advanced grade, according to the Kadish classification (9).

## Therapeutic intervention and follow-up and outcomes

As previous chemotherapy had been unsatisfactory, we sought to apply a more individualized treatment. We performed a next-generation sequencing (NGS) and the result showed a nonsense mutation in the *APC* gene, a tumor suppressor gene involved in the regulation of cell proliferation, migration, adhesion, and chromosomal stability (10) (**Table 1**). *APC* mutation can accelerate the cell cycle, and further promote tumorigenesis and metastasis. Considering that there were no obvious immunotherapeutic target genes detected, we chose to use the broad-spectrum chemotherapeutic agent, MTX, with a novelty way of intrathecal injection.

**Table 1:** Mutations in cancer-related genes.

Category	Chromosome	Position	Gene	Type	AA change	frequency (%)
oncogene	1	156843458	NTRK1	missense mutation	p.E265G	0.6
	1	156843468			p.H268Q	1.3
	1	156843478			p.P272S	0.5
tumor suppressor gene	5	112173798	APC	nonsense mutation	p.S836_S8 37delins	0.5
	19	1221235	STK11	frameshift mutation	p.Y253Sfs34	0.1

Based on these findings, the patient received two cycles of intrathecal MTX injection (10mg, day1, 4, 6) and the intravenous ICE regimen (isocyclophosphamide 1.5g day1-3, etoposide 100mg day1-3, cisplatin 30mg day1-3) since July 2020 (**Figure 2A**). After the first cycle and to reduce the damage and discomfort of each intrathecal injection through a lumbar puncture, we performed Ommaya reservoir implantation in her right frontal brain. The intrathecal injection was performed through the Ommaya reservoir thereafter. The patient was regularly followed up with monthly complete blood count, T and B natural killer cells (TBNK) panel, comprehensive metabolic panel (CMP), and advanced MRI scan.

Due to the severe side effects of isocyclophosphamide, we changed the treatment to intrathecal MTX injection (10mg, day1, 4, 7) and intravenous EC regimen (etoposide 100 mg, day1-4, and carboplatin 100 mg, day1-4) for a total of 3 cycles during October to December 2020. Strikingly, we had a thrilling result of achieving a partial response (PR) and there was an improvement in her physical examination. She became capable of conversing fluently with her healthcare team. She had normal orientation, comprehension, and calculation, with a mildly decreased memory. The grading of power in both upper limbs remained 5. The grading of power was 4 in the right lower limb and 3-4 in the left lower limb. In October 2020, her brain and spine lesions shrunk by ≥ 30% (**Figure 2C**), and in December 2020, her CSF test showed no tumor cells (**Figure 3B**). The proportion of T cells and T helper cells with killing functions increased. The proportion of the Ts cells, which suppress T cells, decreased, further indicating that the treatment had a good regulatory effect on the T cell population(**Figure 3C**).

However, after the third cycle of intrathecal MTX injection and intravenous EC chemotherapy, the patient relapsed on February 2021. She rapidly developed drowsiness and a lack of conversation fluency. Her orientation and comprehension remained normal. However, her calculating capacity and memory were impaired. Her grading of power in the lower limbs decreased, with 2-3 in right lower limb and 3 in the left lower limb. The grading of power of both upper limbs was 5. The MRI showed that the intracranial lesion increased by over 20%, along with cerebral edema (**Figure 2D**). There was also an increase in the percentage of tumor cells in the CSF (**Figure 3B**). When combining the above results with the patient’s symptoms and physical examination, we concluded that she had progressive disease (PD). Although craniospinal irradiation was recommended by our hospital department of radiotherapy, the patient and her family were concerned about the risk of organ damage and bone marrow suppression, and therefore, decided to pursue chemotherapy as a treatment option.

To eliminate the brain edema and better prolong the patient’s survival, bevacizumab was added for four cycles from February to May 2021. During this period, there was no significant difference in her physical examination. Thus, based on these results and the MRI findings (**Figure 2E**), we determined that she reached stable disease (SD). Bevacizumab was stopped in June 2021.

Unfortunately, these approaches failed to further prolong her lifespan. After adjusting to intrathecal MTX injection and intravenous IC regimen with bevacizumab for one cycle, and intrathecal injection of cytarabine (Ara-C) and intravenous IC regimen for another cycle, she passed away in November 2021.

## Discussion

For the past 80 years, only approximately 2000 ONB cases have been reported worldwide (11). The initial symptoms of ONB are non-specific, and according to Xinmao Song, symptoms with the highest to lowest incidence are epistaxis, nasal stenosis, headache, and olfactory dysfunction (12). ONB diagnosis must be histologically confirmed by biopsy (13). However, due to its insidious onset, a definitive diagnosis is typically postponed for 6-12 months, further posing a therapeutic challenge (14). LM is one of the most ominous complications in many cancers, and its incidence in ONB is unclear due to its sparse literature (15). Current studies have employed inconsistent approaches to treat ONB patients and have largely been limited by the sheer number of participants in their studies. The standard treatment remains controversial due to the lacking proof from randomized controlled clinical trials, and therefore, multidisciplinary management is essential. Surgery remains the baseline of treatment and minimally invasive endoscopic resection may be considered to reduce invasiveness (16). Although advanced ONB may respond to chemotherapy, there is no standard chemotherapy regimen for ONB combined with LM (14).

The Ki-67 index is an independent prognostic indicator in ONB, and patients with a higher Ki-67 index have a more unfavorable prognosis (14). Our patient pathological results suggested a high Ki-67 index of 90%, further indicating a high degree of proliferative malignancy. Moreover, the WES suggested a mutation in the *APC* gene which is closely associated with the cell cycle. Therefore, we chose MTX as one of the chemotherapy drugs. MTX is a broad-spectrum anti-tumor drug that plays a vital role in DNA synthesis and primarily acts in rapidly proliferating cells (2, 17). MTX may inhibit the production of dihydrofolate reductase and reduce tetrahydrofolate, both of which are required in DNA synthesis and cell duplication. However, MTX has multiple adverse effects, including bone marrow suppression and organ toxicity in liver, kidney, and lung. Despite these side effects, it is still widely used for the treatment of a variety of malignant diseases due to its high effectiveness.

Considering the brain is protected by the brain-blood-barrier (BBB), we sought to use more effective therapeutic approaches to prolong the patient’s survival with an acceptable quality of life. CSF, the slowly floating liquid that surrounds the brain and the spinal cord, may account for transporting tumor cells to the leptomeninges (18). Although a high intravenous dosage of MTX is often required to obtain an effective dose in the brain microenvironment after crossing the BBB, it is often limited by the unfavorable side effects of nausea, leukopenia, abnormal hepatic function, gastrointestinal ulcer, gastrointestinal hemorrhage, alopecia (19, 20). In contrast, intrathecal injection enables a more direct administration to intracranial lesions and reduces the side effects by reducing MTX dosage. Therefore, we chose to focus on MTX intrathecal administration of MTX.

Another novelty is the use of the Ommaya reservoir. Because this patient required regular intrathecal injections and the lumbar puncture was physically painful for her, the use of the Ommaya reservoir became a more acceptable approach. Ommaya reservoirs are regarded as a secure and safe route of drug delivery that is mainly used for chemotherapy. It has been reported that the Ommaya reservoir implantation may be a good strategy to control intracranial disease symptoms (21). Cancer cells’ infiltration in CSF may block its pathway and lead to malabsorption, resulting in increased intracranial pressure. Ommaya reservoirs can not only relieve intracranial hypertension but also improve the efficacy of drug injection (22). We improve the effectiveness of intrathecal administration by implanting an Ommaya reservoir by extending the patient’s overall survival (OS) to over 15 months. In contrast, the median OS for untreated ONB is only approximately 8 weeks (23).

There are still limitations in our study. Firstly, some clinical information is lacking. Due to the patient’s financial situation, an MRI of the spinal cord could not be perfected after SD (**Figure 2E**). Radiotherapy also made a significant contribution to treating this patient. But as the patient’s family could only provide the information described above, we did not obtain all radiotherapy-related information, including the radiotherapy target volume. A thorough overview of all treatments, including surgery, radiotherapy, chemotherapy, and other methods, is highly recommended and essential to ensure better guidance for future treatment. Secondly, the chemotherapy regimen of systemic chemotherapy and Ommaya reservoir was effective in this patient, but it still needs to be validated in research, including in large sample studies, such as clinical trials, which will direct our future studies.

In conclusion, the findings suggest that intrathecal administration of MTX through the Ommaya reservoir is a promising approach to treating LM arising from a variety of solid and hematologic malignancies.

## Data availability statement

The original contributions presented in the study are included in the article/supplementary materials. Further inquiries can be directed to the corresponding author.

## Ethics statement

Ethical review and approval was not required for the study on human participants in accordance with the local legislation and institutional requirements. The patients/participants provided their written informed consent to participate in this study. Written informed consent was obtained from the individual(s) for the publication of any potentially identifiable images or data included in this article.

## Author contributions

YP and XK contributed to conception and design of the study. BJ, FC, and ZK organized the database. YP wrote the first draft of the manuscript. SY, CW, YL and SL wrote sections of the manuscript. WZ, BZ, JH, and XK proofread and modified the language. WL and FC reviewed and edited the manuscript. All authors contributed to manuscript revision, read, and approved the submitted version.
